# Dengue virus non-structural protein 3 inhibits mitochondrial respiration by impairing complex I function

**DOI:** 10.1128/msphere.00406-24

**Published:** 2024-07-09

**Authors:** Bruna G. Sousa, Nathane C. Mebus-Antunes, Lorena O. Fernandes-Siqueira, Marjolly B. Caruso, Georgia N. Saraiva, Clara F. Carvalho, Thais C. Neves-Martins, Antonio Galina, Russolina B. Zingali, Julianna D. Zeidler, Andrea T. Da Poian

**Affiliations:** 1 Instituto de Bioquímica Médica Leopoldo de Meis, Universidade Federal do Rio de Janeiro, Rio de Janeiro, Brazil; 2 Instituto de Microbiologia Paulo de Góes, Universidade Federal do Rio de Janeiro, Rio de Janeiro, Brazil; Duke-NUS Medical School, Singapore, Singapore

**Keywords:** dengue virus, mitochondrial metabolism, respiratory complexes, non-structural protein 3, viral protease

## Abstract

Dengue virus (DENV) infection is known to affect host cell metabolism, but the molecular players involved are still poorly known. Using a proteomics approach, we identified six DENV proteins associated with mitochondria isolated from infected hepatocytes, and most of the peptides identified were from NS3. We also found an at least twofold decrease of several electron transport system (ETS) host proteins. Thus, we investigated whether NS3 could modulate the ETS function by incubating recombinant DENV NS3 constructs in mitochondria isolated from mouse liver. We found that NS3pro (NS3 protease domain), but not the correspondent catalytically inactive mutant (NS3proS135A), impairs complex I (CI)-dependent NADH:ubiquinone oxidoreductase activity, but not the activities of complexes II, III, IV, or V. Accordingly, using high-resolution respirometry, we found that both NS3pro and full-length NS3 decrease the respiratory rates associated with malate/pyruvate oxidation in mitochondria. The NS3-induced impairment in mitochondrial respiration occurs without altering either leak respiration or mitochondria’s capacity to maintain membrane potential, suggesting that NS3 does not deeply affect mitochondrial integrity. Remarkably, CI activity is also inhibited in DENV-infected cells, supporting that the NS3 effects observed in isolated mitochondria may be relevant in the context of the infection. Finally, *in silico* analyses revealed the presence of potential NS3 cleavage sites in 17 subunits of mouse CI and 16 subunits of human CI, most of them located on the CI surface, suggesting that CI is prone to undergo proteolysis by NS3. Our findings suggest that DENV NS3 can modulate mitochondrial bioenergetics by directly affecting CI function.

**IMPORTANCE:**

Dengue virus (DENV) infection is a major public health problem worldwide, affecting about 400 million people yearly. Despite its importance, many molecular aspects of dengue pathogenesis remain poorly known. For several years, our group has been investigating DENV-induced metabolic alterations in the host cells, focusing on the bioenergetics of mitochondrial respiration. The results of the present study reveal that the DENV non-structural protein 3 (NS3) is found in the mitochondria of infected cells, impairing mitochondrial respiration by directly targeting one of the components of the electron transport system, the respiratory complex I (CI). NS3 acts as the viral protease during the DENV replication cycle, and its proteolytic activity seems necessary for inhibiting CI function. Our findings uncover new nuances of DENV-induced metabolic alterations, highlighting NS3 as an important player in the modulation of mitochondria function during infection.

## INTRODUCTION

Dengue virus (DENV), a member of the *Flaviviridae* family, causes one of the most prevalent tropical infectious diseases worldwide. The viral genome is a single-stranded positive-sense RNA of approximately 11 kb that codes for a polyprotein, which is subsequently processed into three structural proteins (C, prM, and E) and seven non-structural proteins (NS1, NS2A, NS2B, NS3, NS4A, NS4B, and NS5) by host and viral (NS2B-NS3) proteases (1). Dengue virus non-structural proteins participate in multiple steps of DENV replication, the host immune system’s scape, and host cell metabolism modulation (2).

Evidence suggests that DENV infection notably impacts mitochondrial metabolic pathways in the host cell. For instance, fatty acid β-oxidation is induced in the infected cells to provide the energy needed for optimal viral particle production (3–6). In agreement, we have previously demonstrated that, in DENV-infected hepatocytes, glucose is mobilized for anaplerosis to favor mitochondria’s capacity for oxidizing fatty acids, while glutamine oxidation is inhibited (7). However, although we have shown that different parameters of mitochondrial bioenergetics are affected during DENV infection in distinct cellular models (2, 7–10), it was still unknown whether infection could directly impact the activity of mitochondrial respiratory complexes at a molecular level.

Here, we performed a proteomic screening in isolated mitochondria from DENV-infected hepatocytes. The analyses showed six DENV proteins associated with the mitochondria, with most of the peptides identified belonging to NS3. DENV NS3 is a multi-functional protein with approximately 70 kDa, formed by three structural domains comprising different enzymatic activities, namely the N-terminal trypsin-like serine protease domain (NS3pro), the nucleoside triphosphatase (NTPase) domain, and the C-terminal helicase and RNA 5′-triphosphatase domains (11–14). Thus, besides processing the viral polyprotein, NS3 participates in viral RNA replication (unwinding step) and capping. Additionally, it has been shown that NS3 interacts with and stimulates the activity of fatty acid synthase (FASN), redistributing FASN to sites of viral replication and increasing fatty acid synthesis locally (15, 16). Furthermore, it has recently been shown that DENV NS2B-NS3 migrates to the nucleus, where it cleaves EDRF1, a transcriptional factor involved in platelet formation (17). At the same time, the NS3pro (without the NS2B cofactor) is imported into the mitochondrial matrix, where it cleaves the protein GrpEL1, a mitochondrial Hsp70 co-chaperon (17, 18). All these findings show that DENV NS3 plays multiple key roles in viral replication, being considered one of the most attractive targets for DENV antiviral therapy (19).

In this context, we investigated the mitochondrial bioenergetics and the specific function of the electron transport system (ETS) protein complexes in isolated mouse liver mitochondria incubated with recombinant DENV NS3 protein constructs. We found that NS3 impairs complex I (NADH:ubiquinone oxidoreductase) activity likely in a protease-dependent manner, but not complex II (succinate dehydrogenase), complex III (ubiquinone/cytochrome *c* oxidoreductase), complex IV (cytochrome *c* oxidase) or complex V (ATP synthase) activities. Accordingly, NS3 impairs the oxidation of substrates that generate NADH in mitochondria, such as malate and pyruvate. Similarly, CI activity is also inhibited in DENV-infected cells, suggesting that the results obtained with isolated mitochondria may also be relevant in the context of the infection. These findings shed light on the possible mechanism by which DENV NS3 targets mitochondrial metabolism in infected cells.

## RESULTS

### DENV NS3 localizes in the mitochondria of infected cells

**Fig 1:**
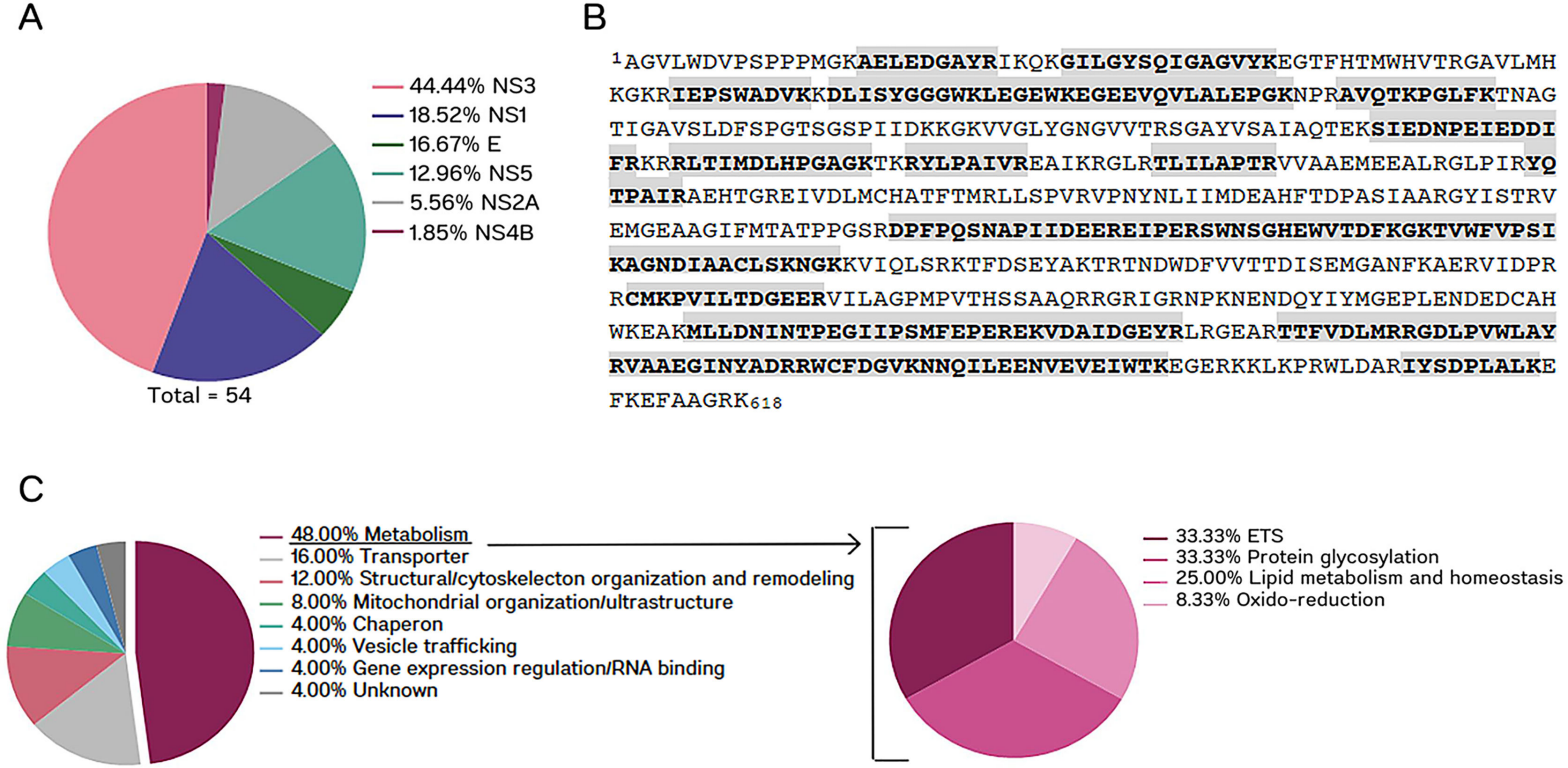
Proteomics screening of isolated mitochondria from DENV-infected Huh7 cells. (**A**) Pie chart showing the percentage of DENV peptides identified by mass spectrometry. (**B**) NS3 sequence, indicating the coverage of the peptides identified by mass spectrometry (highlighted in gray), which corresponded to 46.60% of the protein (288 out of 618 aa). Uniprot information: Entry: P29990; Entry name: POLG_DEN26; Protein name: Genome polyprotein; Organism: Dengue virus type 2 (strain Thailand/16681/1984) (DENV-2); Length: 3391 aa. (**C**) Pie charts with the downregulated proteins (at least twofold) in the mitochondria of DENV-infected cells relative to mock, categorized by function (according to the UniProt database). ETS, electron transport system.

We and others have shown that DENV infection affects mitochondrial function in the host cells, altering the preference for oxidizing energetic substrates in this organelle (2, 7, 8). To better understand the viral molecular players that mediate these effects, we performed a proteomic analysis of mitochondria isolated from DENV-infected Huh7 cells. In this screening, we identified peptides from six DENV proteins in the mitochondria preparation, namely E, NS1, NS2A, NS3, NS4A, and NS5 (Table 1). Most of the viral peptides identified by mass spectrometry belonged to NS3 (44.4%), which covered 46.6% of the whole protein, spanning both protease and helicase domains (Fig. 1A and B). We also noticed that about half of the proteins that appear in lower abundance in the mitochondria of infected cells (at least twofold compared to mock control) were related to metabolism, according to the function classification in the UniProt database (Fig. 1C). Of these, one-third belonged to the mitochondrial electron transport system (Fig. 1C; Table S1). Thus, we hypothesized that NS3 could directly interfere with the mitochondrial respiration of the host cells.

**TABLE 1:** DENV peptides identified through mass spectrometry of isolated mitochondria from Huh7 cells

Peptide sequence	Peptide length (aa)	Protein
AELEDGAYR	9	Serine protease NS3 (protease domain)
GILGYSQIGAGVYK	14	Serine protease NS3 (protease domain)
IEPSWADVK	9	Serine protease NS3 (protease domain)
DLISYGGGWK	10	Serine protease NS3 (protease domain)
LEGEWKEGEEVQVLALEPGK	20	Serine protease NS3 (protease domain)
AVQTKPGLFK	10	Serine protease NS3 (protease domain)
SIEDNPEIEDDIFR	14	Serine protease NS3 (protease domain)
RLTIMDLHPGAGK	13	Serine protease NS3 (helicase domain)
RYLPAIVR	8	Serine protease NS3 (helicase domain)
TLILAPTR	8	Serine protease NS3 (helicase domain)
YQTPAIR	7	Serine protease NS3 (helicase domain)
DPFPQSNAPIIDEEREIPER	20	Serine protease NS3 (helicase domain)
SWNSGHEWVTDFKGK	15	Serine protease NS3 (helicase domain)
TVWFVPSIK	9	Serine protease NS3 (helicase domain)
AGNDIAACLSKNGK	14	Serine protease NS3 (helicase domain)
CMKPVILTDGEER	13	Serine protease NS3 (helicase domain)
MLLDNINTPEGIIPSMFEPEREK	23	Serine protease NS3 (helicase domain)
EKVDAIDGEYR	11	Serine protease NS3 (helicase domain)
TTFVDLMRR	9	Serine protease NS3 (helicase domain)
RGDLPVWLAYR	11	Serine protease NS3 (helicase domain)
VAAEGINYADRR	12	Serine protease NS3 (helicase domain)
WCFDGVK	7	Serine protease NS3 (helicase domain)
NNQILEENVEVEIWTK	16	Serine protease NS3 (helicase domain)
IYSDPLALK	9	Serine protease NS3 (helicase domain)
CGSGIFITDNVHTWTEQYK	19	Non-structural protein 1
AHEEGICGIR	10	Non-structural protein 1
LENLMWK	7	Non-structural protein 1
QITPELNHILSENEVK	16	Non-structural protein 1
LTIMTGDIK	9	Non-structural protein 1
SLRPQPTELK	10	Non-structural protein 1
ASFIEVK	7	Non-structural protein 1
SHTLWSNGVLESEMIIPK	18	Non-structural protein 1
NLAGPVSQHNYRPGYHTQITGPWHLGK	27	Non-structural protein 1
LITEWCCR	8	Non-structural protein 1
NKPTLDFELIK	11	Envelope protein E
CPTQGEPSLNEEQDKR	16	Envelope protein E
HSMVDRGWGNGCGLFGK	17	Envelope protein E
GGIVTCAMFR	10	Envelope protein E
ITPQSSTTEAELTGYGTVTMECSPR	25	Envelope protein E
ETLVTFK	7	Envelope protein E
LITVNPIVTEK	11	Envelope protein E
DSPVNIEAEPPFGDSYIIIGVEPGQLK	27	Envelope protein E
GSSIGQMFETTMR	13	Envelope protein E
LQSGVDVFFIPPEK	14	RNA-directed RNA polymerase NS5
NIGIESEIPNLDIIGK	16	RNA-directed RNA polymerase NS5
EAVEDSRFWELVDK	14	RNA-directed RNA polymerase NS5
GSRAIWYMWLGAR	13	RNA-directed RNA polymerase NS5
EGGAMYADDTAGWDTR	16	RNA-directed RNA polymerase NS5
TPVESWEEIPYLGK	14	RNA-directed RNA polymerase NS5
EDQWCGSLIGLTSR	14	RNA-directed RNA polymerase NS5
VRPTFAAGLLLR	12	Non-structural protein 2A
TDWIPLALTIK	11	Non-structural protein 2A
GLNPTAIFLTTLSR	14	Non-structural protein 2A
NPTVDGITVIDLDPIPYDPK	20	Non-structural protein 4B

### NS3pro, but not NS3proS135A, inhibits CI activity

To investigate whether DENV NS3 interferes with the function of respiratory complexes, here we used three constructs of the recombinant NS3: the protease domain (NS3pro, residues 1–180); a mutant of this domain containing the serine residue of the catalytic site replaced by an alanine residue (NS3proS135A); and the full-length protein (NS3prohel, residues 1–618) (Fig. S1A through C). Since the NS3 protease domain was shown to be imported into mitochondria more efficiently than the full-length protein (18), we used NS3pro and the catalytically inactive mutant in our first set of experiments, whose results were then confirmed using the full-length protein or in the context of infection. Although it has been shown that the *in vitro* NS3 proteolytic activity against a viral natural substrate (NS4B-NS5 precursor) depends on the NS2B co-factor (20), using a fluorogenic substrate for serine proteases, we confirmed that NS3pro displays proteolytic activity alone, which is highly decreased in the mutant protein (Fig. S1D).

To address whether NS3pro could directly impair the activity of each ETS complex, we incubated isolated mitochondria from mouse liver with recombinant NS3pro or NS3proS135A for 1 h on ice before assaying the activities of complex I (CI; NADH:ubiquinone oxidoreductase), complex II (CII; succinate dehydrogenase), complex III (CII; ubiquinone/cytochrome *c* oxidoreductase), complex IV (CIV; cytochrome *c* oxidase), and complex V (CV; ATP synthase).

**Fig 2:**
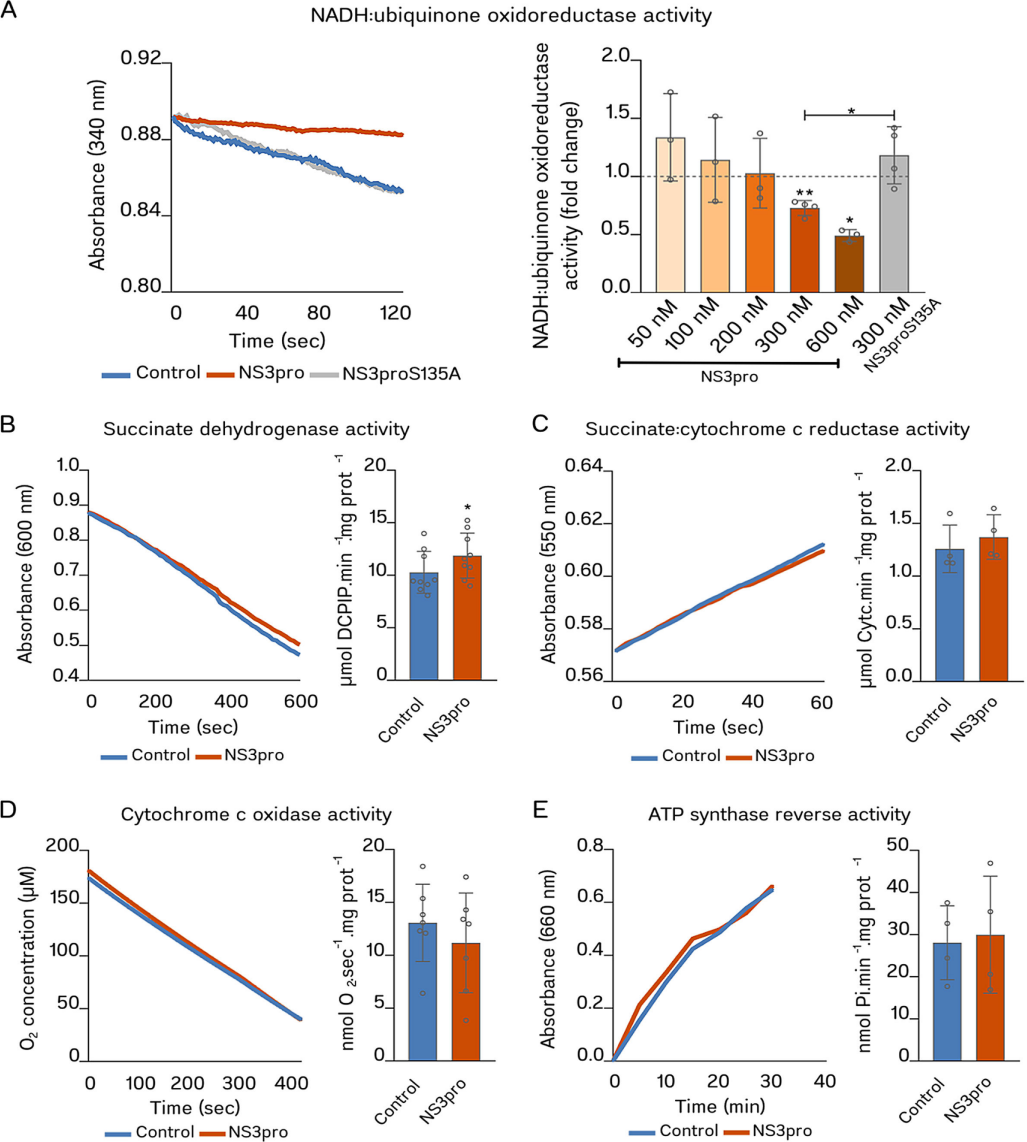
NS3pro inhibits CI, but not CII, CIII, CIV, or CV activities. Protein extracts from isolated mitochondria pre-incubated with 300 nM NS3pro or NS3proS135A (when indicated) were assayed for: (**A**) NADH:ubiquinone oxidoreductase activity (*n* = 3–4); (**B**) succinate dehydrogenase activity (*n* = 9); (**C**) succinate:cytochrome *c* reductase activity (CII + CIII) (*n* = 4); (**D**) cytochrome *c* oxidase activity (*n* = 7); and (**E**) ATP synthase reverse activity (*n* = 4). In all panels, the graph on the left shows a representative kinetics plot, whereas the graph on the right represents the reaction rates obtained from independent experiments. Here and in all other figures, the control condition was represented in blue, NS3pro in orange, and NS3proS135A mutant in gray. In (**A**), the light-to-dark orange gradient represents increasing concentrations of NS3pro. Data expressed as mean ± standard deviation. Significant differences between means were analyzed using the Student’s *t* test. *, *P* < 0,05; **, *P* < 0.01.

To measure CI activity, we provided NADH (electron donor) and ubiquinone Q_1_ (electron acceptor) to the mitochondria preparation and measured the rotenone (CI inhibitor)-dependent NADH consumption photometrically (Fig. 2A). Our results showed that mitochondria incubation with NS3pro decreases NADH-ubiquinone oxidoreductase activity in a dose-dependent manner, reaching significant inhibition rates of 26.9% (S.D. ± 6.4%, *P* = 0.0025) or 50.6% (S.D. ± 5%, *P* = 0.0123) at 300 nM or 600 nM NS3pro concentrations, respectively. In contrast, CI activity in mitochondria incubated with NS3proS135A was not significantly different from that observed for the control sample. This result indicates that NS3pro directly inhibits CI activity, likely in a protease-dependent manner.

To assay CII activity, we provided succinate (the electron donor) and oxidized DCPIP (the electron acceptor) to a reaction mix containing mitochondria previously incubated with NS3pro (Fig. 2B). In this assay, succinate oxidation is traced by measuring the formation of reduced DCPIP spectrophotometrically. With this approach, we observed that NS3pro not only did not impair CII activity but slightly stimulated it (an increase of 15.6%, S.D. ± 20.9%, *P* = 0.0124), indicating that NS3pro does not inhibit CII activity directly.

Since NS3pro pre-incubation does not impair CII activity, we performed CII + CIII combined activities to indirectly address CIII (ubiquinone/cytochrome *c* oxidoreductase) function in this condition. For this, we provided succinate as an electron donor and cytochrome *c* as the electron acceptor to a reaction media containing mitochondria samples (Fig. 2C). Cytochrome *c* oxidation was then measured spectrophotometrically. Since CII + CIII activity was not significantly altered by NS3pro pre-incubation, by exclusion, we assumed that NS3pro does not affect mitochondrial CIII activity.

To address CIV activity, we measured the oxygen consumption of mitochondria samples in the presence of reduced TMPD, a CIV-specific electron donor (Fig. 2D). The oxygen consumption rates (OCRs) of NS3pro pre-incubated mitochondria were similar to those of control mitochondria. Therefore, our data suggest that NS3pro does not significantly affect CIV activity.

Finally, we measured CV (ATP synthase) activity in mitochondria incubated with NS3pro. By taking advantage of its remarkable feature of operating in the reverse direction when the electrochemical gradient is low (expected in the conditions of our experiment, where mitochondrial membranes are disrupted) (21), we evaluated the azide (an ATP synthase inhibitor)-sensitive fraction of the ATP synthase reverse activity (ATP hydrolysis) by providing ATP to the reaction media and measuring the formation of inorganic phosphate. In this assay, we did not observe significant differences in CV activity in mitochondria preparations incubated with NS3pro compared to control (Fig. 2E), suggesting that NS3pro does not interfere with its function directly.

In summary, our data indicate that NS3pro can directly impair CI activity but not the activity of the other respiratory complexes in mitochondria.

### NS3pro inhibits pyruvate/malate utilization in mitochondria

**Fig 3:**
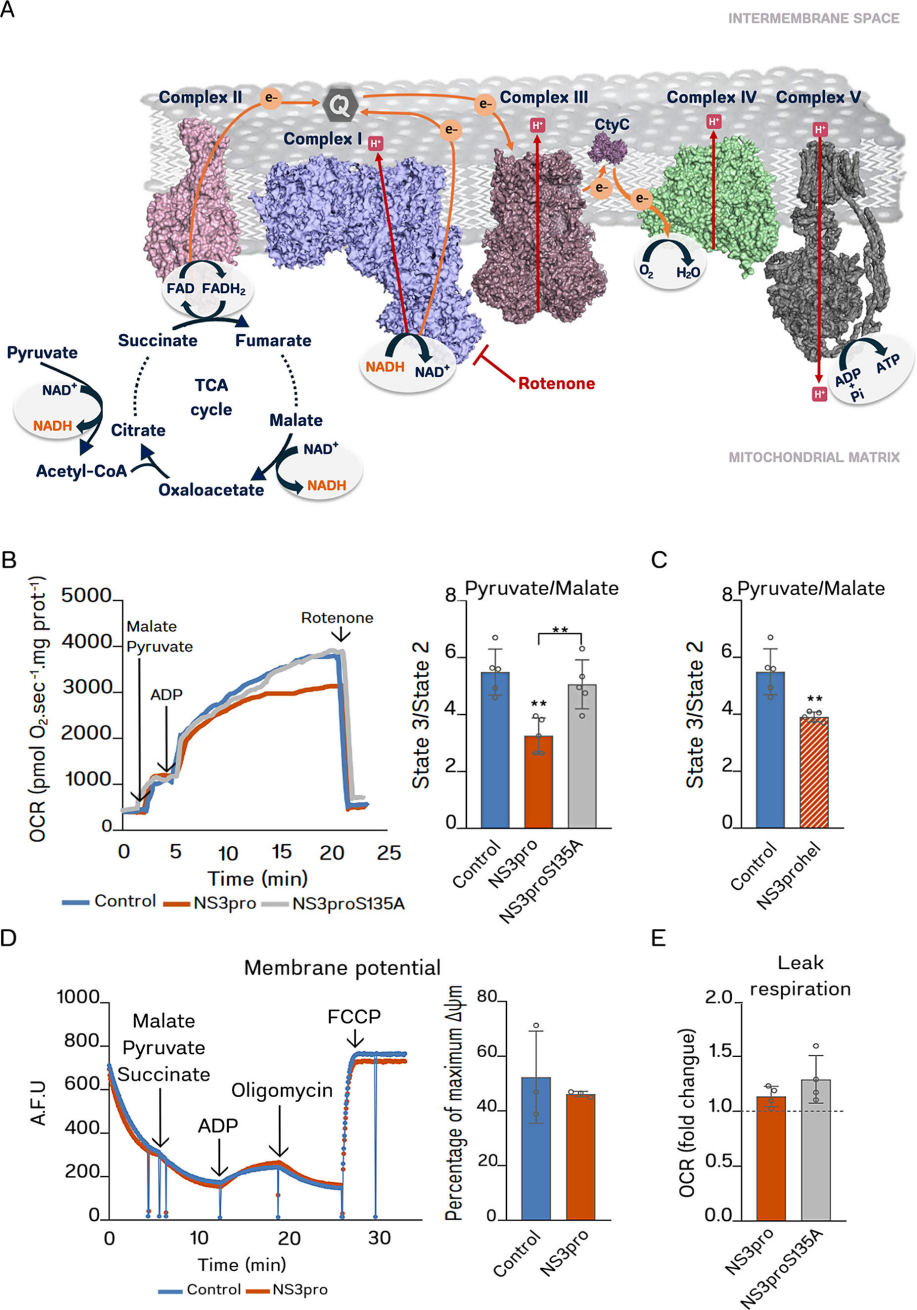
NS3pro affects malate/pyruvate oxidation in mitochondria. (**A**) Illustrative scheme of the electron transport system (ETS). Pyruvate and malate oxidation (in dark blue) generate NADH (in orange), whose electrons flow through CI. These substrates and the CI inhibitor rotenone (in red) were used in this figure’s respirometry experiments. The dotted line in the tricarboxylic acid (TCA) cycle, as well as the reactions in light gray, do not occur significantly in the condition of the experiments since citrate is exported from mitochondria in exchange for malate (which is in high concentration) through a tricarboxylate carrier. Electrons (e−) were represented in orange circles; protons (H^+^) in red squares; and ubiquinone (**Q**) in a gray hexagon. Flat-tipped arrows in red indicate inhibition. The structures of the respiratory complexes were generated with PyMOL using PDB IDs 5XTD (CI), 1NTM (CIII), 3ZCF (cytochrome *c*), 2Y69 (CIV), and 5ARE (CV–ATP synthase). (**B and C**) High-resolution respirometry experiments with isolated mitochondria incubated with (**B**) NS3pro or NS3proS135A (*n* = 5); and (**C**) full-length NS3 (NS3prohel, bar with orange diagonal stripes, *n* = 5), using pyruvate and malate as substrates followed by the sequential addition of ADP and the CI inhibitor rotenone, as indicated. In (**B**), on the left, a representative high-resolution respirometry experiment and, on the right, fold change of respiratory state 3 relative to respiratory state 2 in independent experiments (*n* = 5). The residual non-mitochondrial oxygen consumption rate obtained with rotenone was discounted from the analyses. (**D**) On the left, representative curves for mitochondrial membrane potential measured by safranin O fluorescence self-quenching in a multi-substrate experiment with isolated mitochondria incubated or not with NS3pro (300 nM). The additions of substrates and inhibitors are indicated in the figure. The graph on the right shows the percentage of the maximal ΔΨm in independent experiments (*n* = 3). (**E**) Proton leak respiration of isolated mitochondria incubated with 300 nM NS3pro or NS3proS135A (*n* = 4), represented as fold change relative to control. Significant differences between means were analyzed using one-way ANOVA with Sidak’s post hoc test (**B**) or Student’s *t* test (**C–E**). All data were expressed as mean ± standard deviation. **, *P* < 0.01.

To evaluate the effects of NS3 on CI activity in the context of mitochondrial respiratory function, we performed high-resolution respirometry experiments. In these experiments, we used the NADH-generating substrates pyruvate and malate to feed mitochondrial ETS at CI, as represented in Fig. 3A. Electrons coming from NADH flow through CI to ubiquinone to reach CIII, cytochrome *c*, and CIV, to finally reduce oxygen to water. Electron transport through CI, CIII, and CIV is coupled to H^+^ pumping to the mitochondrial intermembrane space, generating an electrochemical gradient that drives ATP synthesis as the H^+^ returns to the mitochondrial matrix through ATP synthase. At first glance, one could expect that malate and pyruvate form succinate due to their metabolization in the Krebs cycle, thus indirectly feeding CII (see Fig. 3A). However, the high activity of the tricarboxylate carrier in liver mitochondria causes citrate to be lost from the mitochondria in exchange for malate before it can be oxidized in the cycle (22), ensuring that the results presented here reflect only CI-dependent respiratory activity.

To evaluate the effects of NS3 on the mitochondrial function associated with CI activity, we pre-incubated mitochondria samples with NS3pro or NS3proS135A and measured the OCR using pyruvate and malate as respiratory substrates. Representative OCR curves are shown in Fig. 3B (left panel). OCR value obtained after adding malate and pyruvate reflects mitochondrial respiration unrelated to ATP synthesis, as ADP (the substrate for ATP synthesis) is absent in the medium. This condition is referred to as state 2 respiration. The increase in oxygen consumption observed after the addition of ADP (the difference between OCR values before and after ADP addition) corresponds to the mitochondrial respiration coupled to the ATP synthesis. The OCR value reached after ADP addition, which represents the ETS physiological functioning (with all substrates), is defined as state 3 respiration. The addition of rotenone, an inhibitor of CI, provides the non-mitochondrial OCR value, which is discounted in the analyses. To normalize the variances of the raw data, here we represented the respirometry data as the fold change in state 3 relative to state two respiration.

We found that mitochondrial respiration associated with pyruvate/malate decreases by 40.2% (S.D. ± 10.9%, *P* = 0.0035) in mitochondria incubated with NS3pro but not with NS3proS135A, indicating that NS3pro interferes with pyruvate/malate-associated oxygen consumption probably in a protease-dependent manner (Fig. 3B, right panel). Similarly, incubation of mitochondria with the full-length NS3 construct, NS3prohel, promoted a 28.4% decrease in pyruvate/malate-dependent respiration (S.D. ± 3.2%, *P* = 0.0026) (Fig. 3C), showing that this effect is also observed using the native protein and, thus, can occur in the context of the infection. To rule out an eventual unspecific effect of other DENV proteins on CI function, we performed a similar experiment preincubating a recombinant DENV capsid (C) protein preparation with mitochondria. We found that, unlike NS3pro, DENV C protein does not significantly change pyruvate/malate-associated respiration (Fig. S2).

To ensure the integrity of the mitochondria preparation after incubation with NS3pro, we analyzed the mitochondrial membrane potential (ΔΨm) and proton leak after incubation with NS3pro at 300 nM concentration in a multi-substrate protocol (with pyruvate, malate, and succinate feeding ETS simultaneously through CI and CII). We measured the mitochondrial capacity to maintain membrane potential using safranin O in a fluorescence quenching assay. In this assay, oligomycin (an ATP synthase inhibitor that impairs the flow of H^+^ back to the matrix) and FCCP (a proton ionophore) are used to determine the maximum and minimum membrane potential, respectively (Fig. 3D). Then, we measured the mitochondrial membrane potential (ΔΨm) in the phosphorylating state by calculating the percentage of the maximum ΔΨm maintained after ADP addition. We found that incubation with NS3pro did not significantly affect ΔΨm in this condition (Fig. 3D), confirming that these mitochondria keep the capacity to maintain membrane potential. Also, the percentage of the ΔΨm depolarized after ADP addition falls in the physiological range of 15%–20% described by Starkov et al. (23). Thus, the decrease in coupled respiration induced by 300 nM NS3pro seems to be due to some impairment in the mitochondrial metabolism rather than an increase in the intrinsic mitochondrial uncoupling or an extrinsic pathological dyscoupling (which could happen due to membrane disruption or other mechanisms of proton leaking). Indeed, we found that NS3pro does not alter the leak respiration (a non-phosphorylating resting state when reducing substrates but not ADP is provided) (Fig. 3E), suggesting that mitochondria were still coupled in this condition.

### Complex I activity is impaired in mitochondria from DENV-infected cells

**Fig 4:**
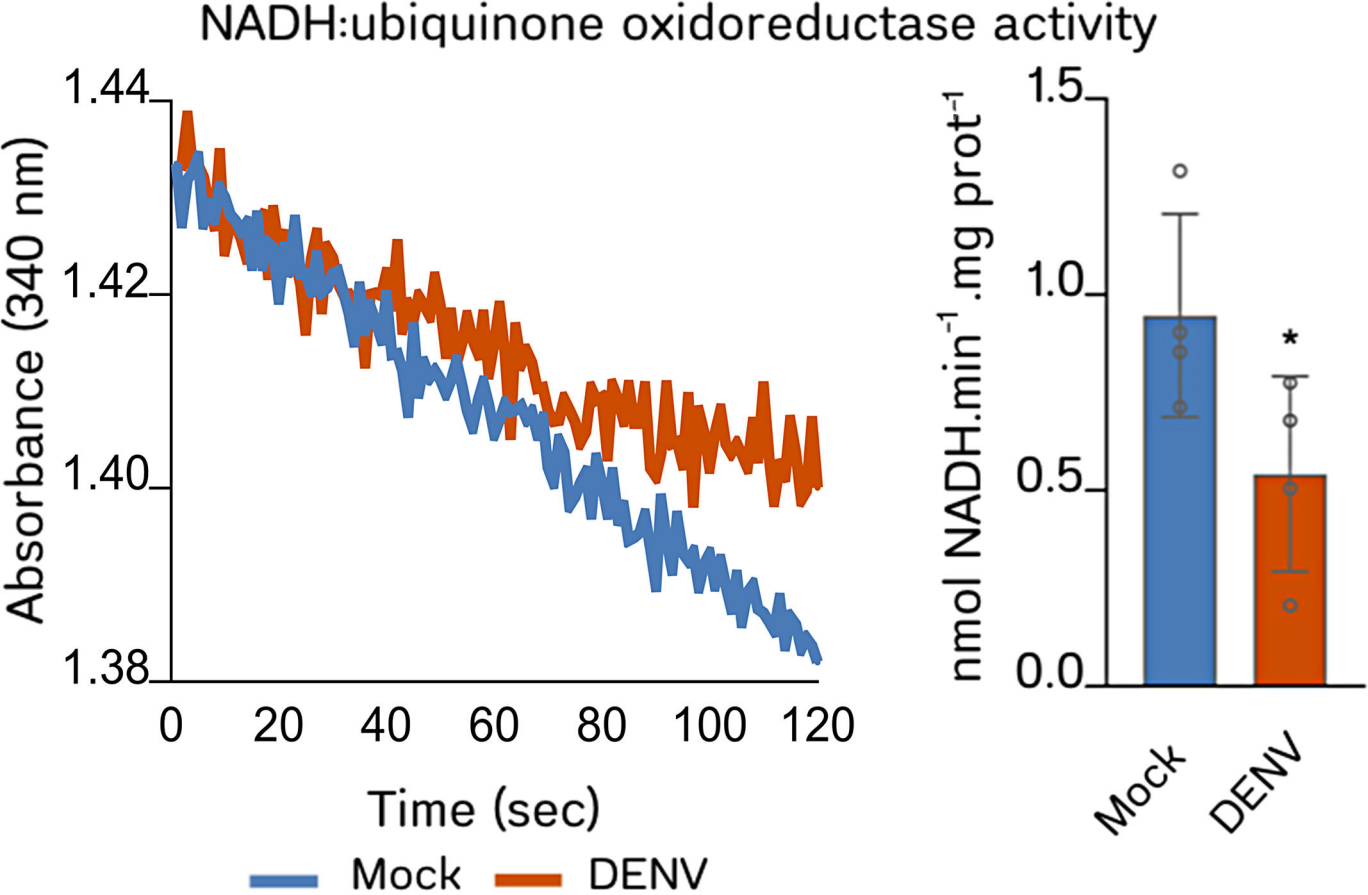
DENV-infected Huh7 cells have impaired CI activity. Protein extracts from Huh7 cells infected with DENV (serotype 2, strain 16681, MOI = 1) or cells subjected to simulated infection (mock) were assayed for NADH:ubiquinone oxidoreductase activity. The graph on the left shows representative kinetics in the different conditions, whereas the graph on the right shows the reaction rates obtained in independent experiments (*n* = 4). Data expressed as mean ± standard deviation. Significant differences between means were analyzed using Student’s *t* test. *, *P* = 0.0162.

Since all the experiments shown so far were performed in isolated mitochondria, we wondered whether CI activity is also impaired in the mitochondria of DENV-infected cells. For this, we infected Huh7 cells with DENV [multiplicity of infection (MOI) = 1] and analyzed CI activity in cell lysates harvested 24 h post-infection (hpi). We observed that CI activity is 44.15% (S.D. ± 20.69%, *P* = 0.0162) lower in the samples of Huh7-infected cells compared to the mock control (Fig. 4). These data indicate that NS3 may mediate this inhibitory effect on CI activity also in the context of DENV infection.

### Complex I subunits contain multiple predicted NS3 cleavage sites

Since the proteolytic activity of NS3pro seems to be required to inhibit CI activity, we searched for potential NS3 cleavage sites in CI subunits. For this, we performed an *in silico* analysis using the SitePrediction website, in which one informs the protease candidate substrates as inputs (24). The NS2B-NS3 cleavage motifs present in flavivirus polyproteins consist of a pair of basic amino acid residues (KR, RR, RK, or occasionally QR) at the canonical positions P2 and P1 followed by a small amino acid (G, S, or A) in position P1′ (25, 26). Since NS3 protease is not included in the SitePrediction database, we entered all the 12 amino acid combinations that create potential cleavage sites at positions P2-P1xP1′ (KRA, KRG, KRS, RRA, RRG, RRS, RKA, RKG, RKS, QRA, QRG, and QRS). As substrate inputs, we entered both mouse (since the results were obtained using mouse mitochondria) and human (to get insights on the applicability of the results in the context of dengue pathogenesis) protein sequences of the 44 CI subunits. It is important to mention that mouse and human CI subunits show high sequence identity, ranging from 56% to 96% considering all the 44 subunits and 75%–96% considering those containing potential cleavage sites in both species.

The analyses revealed the presence of potential NS3 cleavage sites in 17 subunits of mouse CI and 16 subunits of human CI structure (Table 2; Fig. S3A). Among them, one subunit, NDUFS1, contains three potential cleavage sites both in mouse and human sequences, while the NDUFV1 subunit contains two potential cleavage sites in mouse and one in human, and NDUFB4 contains two potential cleavage sites in human and one in mouse sequence. The NDUFB5 subunit contains two potential cleavage sites only in mouse sequence. Therefore, 21 and 19 potential NS3 cleavage sites occur in the mouse and human CI structures, respectively. To evaluate whether these potential DENV NS3 cleavage sites are exposed on the surface of the assembled CI structure, we highlighted them on the cryoEM structure of human CI. We found that the predicted cleavage sites in NDUFS1, NDUFS2, NDUFV1, NDUFA2, NDUFA12, NDUFA13, NDUFB1, and NDUFB4 subunits are in more exposed regions of the CI structure (Fig. S3B). Therefore, our analyses suggest that CI is prone to undergo proteolysis by DENV NS3.

**TABLE 2:** Predicted NS3 protease cleavage sites in mouse and human mitochondrial CI subunits^*a*^

Complex I subunit		Abbreviation	UniProt (mouse/human)	Cleavage site sequence	Cleavage site position	Identification in Fig. S3
NADH-ubiquinone oxidoreductase chain 1		NU1M	P03888/P03886	RKG	34–36	A
NADH-ubiquinone oxidoreductase chain 4		NU4M	P03911/P03905	QRG (only M)	415–417	-
NADH dehydrogenase (ubiquinone) Fe-S protein 1		NDUFS1	Q91VD9/P28331	KRA/RKA (M/H) KRA RKS	7–9 157–159 419–421	B
NADH dehydrogenase (ubiquinone) Fe-S protein 2		NDUFS2	Q91WD5/O75306	KRA	367–369	C
NADH dehydrogenase (ubiquinone) Fe-S protein 4		NDUFS4	Q9CXZ1/O43181	RRA	17–19	-
NADH dehydrogenase (ubiquinone) Fe-S protein 5		NDUFS5	Q99LY9/O43920	RRA (only H)	74–76	D
NADH dehydrogenase (ubiquinone) Fe-S protein 7		NDUFS7	Q9DC70/O75251	RRS	86–88/75–77 (M/H)	E
NADH dehydrogenase (ubiquinone) flavoprotein 1		NDUFV1	Q91YT0/P49821	RRG (only M) RRG	58–60 256–258	F
NADH dehydrogenase (ubiquinone) 1 alpha subcomplex subunit 2		NDUFA2	Q9CQ75/O43678	QRS	25–27	G
NADH dehydrogenase (ubiquinone) 1 alpha subcomplex subunit 9		NDUFA9	Q9DC69/Q16795	RKA (only M)	117–119	-
NADH dehydrogenase (ubiquinone) 1 alpha subcomplex subunit 11		NDUFA11	Q9D8B4/Q86Y39	RKA (only H)	20–22	H
NADH dehydrogenase (ubiquinone) 1 alpha subcomplex subunit 12		NDUFA12	Q7TMF3/Q9UI09	KRG	8–10	I
NADH dehydrogenase (ubiquinone) 1 alpha subcomplex subunit 13		NDUFA13	Q9ERS2/Q9P0J0	RRG	27–29	J
NADH dehydrogenase (ubiquinone) 1 beta subcomplex subunit 1		NDUFB1	P0DN34/O75438	RKS (only H)	29–31	K
NADH dehydrogenase (ubiquinone) 1 beta subcomplex subunit 4		NDUFB4	Q9CQC7/O95168	RRA RRG (only H)	30–32 56–58	L
NADH dehydrogenase (ubiquinone) 1 beta subcomplex subunit 5		NDUFB5	Q9CQH3/O43674	QRA (only M) RRA (only M)	8–10 21–23	--
NADH dehydrogenase (ubiquinone) 1 beta subcomplex subunit 7		NDUFB7	Q9CR61/P17568	RRA (only M)	114–116	-
NADH dehydrogenase (ubiquinone) 1 beta subcomplex subunit 8		NDUFB8	Q9D6J5/O95169	QRA (only H) RRA (only M)	15–17 27–29	-
NADH dehydrogenase (ubiquinone) 1 beta subcomplex subunit 9		NDUFB9	Q9CQJ8/Q9Y6M9	KRA	21–23	M
NADH dehydrogenase (ubiquinone) 1 beta subcomplex subunit 10		NDUFB10	Q9DCS9/O96000	RKA	167–169/161–163 (M/H)	N

^*a*^ M: site found in mouse ; H: site found in human; (-) cleavage site not possible to be represented the in the structure.

## DISCUSSION

DENV infection is known to elicit a variety of metabolic alterations in the host cells, including changes in the preference for oxidizing energetic substrates in mitochondria (2, 7). Here, we show that DENV NS3 impairs CI activity directly, likely by promoting proteolytic cleavage of CI subunits. Accordingly, we also demonstrate that NS3 affects mitochondria’s capacity to utilize malate and pyruvate as respiratory substrates, which feeds the ETS by generating NADH, the CI substrate. These results agree with our previous findings demonstrating that the main respiratory substrate used in DENV-infected cells shifts from glutamine to palmitate (7), as electrons from fatty acids feed ETS from acyl-CoA dehydrogenase/electron transfer flavoprotein (ETF)/electron flavoprotein dehydrogenase (ETFDH) directly to ubiquinone, thus bypassing CI. Therefore, we suggest that DENV NS3 directly interferes with the mitochondrial ETS capacity in a substrate-specific manner and may be one of the molecular players that mediate the metabolic alterations in DENV-infected cells.

NS3 is a trypsin-like serine protease highly conserved among flavivirus (27). During viral polyprotein processing, flavivirus NS3 protease is believed to be fully active when interacting with its cofactor NS2B (12, 20, 26). However, NS2B was not found among the six DENV proteins we identified in the mitochondrial fraction of infected cells (NS3, NS1, NS2A, NS4A, NS5, and E). Nonetheless, it is possible that NS3 binding to specific host substrates allows conformational changes that favor its activity irrespective of NS2B. In agreement, recent studies showed that NS3pro alone can cleave the mitochondrial Hsp70 co-chaperone GrpEl1 after being imported to isolated mouse liver mitochondria (17, 18). In these studies, the authors also demonstrated that NS3pro, but not NS3proS135A (the same construct we used here for the catalytically inactive NS3), cleaved recombinant GrpEl1 *in vitro*, confirming that NS3pro alone possesses proteolytic activity for this substrate. Likewise, an *in vitro* assay showed that NS3 alone has proteolytic activity when chromogenic substrates containing arginine residues were used (28), which we confirmed for the recombinant NS3pro protein preparations used in the present study.

After being cleaved from the viral polyprotein, NS2B remains an integral ER membrane protein. In this context, NS2B was shown to be essential for recruiting Zika virus (also a flavivirus) NS3 to the viral replication complex (29). Although most DENV NS3 associates with the ER during infection (30), it possesses a mitochondrial import signal, allowing it to be imported into the mitochondrial matrix (18). Accordingly, NS3 is enriched in the mitochondria fraction of DENV-infected cells (31, 32), and NS3-transfected cells display decreased mitochondrial respiratory rates (17). These studies support that NS3 is also addressed to mitochondria, where it can play other functions besides its role as part of the viral replication complex. Our findings here are the first to demonstrate that NS3 can modulate mitochondrial metabolism by directly interfering with the function of an ETS complex.

CI is the largest and most intricate mitochondrial respiratory complex (33). It transfers electrons from NADH to ubiquinone in a process coupled with the translocation of protons across the inner mitochondrial membrane. The mammalian CI has 44 subunits that are organized into three functional modules: the N module (NADH oxidation module), the Q module (ubiquinone reduction module), and the P module (proton pump module), the later constituting the arm embedded in the mitochondria inner membrane (33). Among the subunits within the CI structure, 14 are considered core subunits, which play active roles in CI enzymatic activity, while the remaining 30 are accessory subunits (33, 34). Mutations in all core subunits and 12 of the accessory subunits have already been described in human diseases associated with CI deficiency (34).

Here, we showed that 16 subunits of human CI (or 17 subunits in mice CI) possess potential DENV NS3 cleavage sites. Among these cleavage sites, those located in NDUFS1, NDUFS2, NDUFV1, NDUFA2, NDUFA12, NDUFA13, NDUFB1, and NDUFB4 subunits are more exposed on the CI surface, presumably being more accessible for proteolysis by NS3. NDUFS1 and NDUFS2 are core subunits, and mutations in the accessory subunits NDUFV1, NDUFA2, NDUFA12, and NDUFA13 were associated with human diseases (34). Therefore, the cleavage of one or more subunits during DENV infection may affect CI function. It does not mean, however, that subunits localized more internally in the CI structure are not accessible to NS3 since the interaction NS3/CI could expose regions normally hidden in the native CI structure. Thus, the proteolysis of the more internally localized NDUFS1, NDUFS7, and NDUFV1 subunits, which possess iron-sulfur centers and are essential to the electron transport within the CI structure, could also dramatically impair CI function.

A caveat in our data is that we cannot guarantee that the effects of NS3pro on CI activity depend on proteolysis of CI subunits since we did not demonstrate it directly. Even though NS3proS135A contains a mutation in the catalytic amino acid triad and has impaired activity, the mutation may disturb the protein conformation, thus preventing a presumably protease-independent inhibition of CI. Therefore, more evidence is required to confirm whether some of the 44 CI subunit is indeed a target of DENV NS3 proteolysis. We aim to focus on that in a future study.

Other DENV proteins were shown to localize in mitochondria. One example is the NS4B protein, which inhibits the activation of the mitochondrial fission factor DRP1, inducing mitochondrial elongation and, thus, favoring the oxidative metabolism in infected cells (31, 32). Our data suggest NS3 may also contribute to metabolic reprogramming during DENV infection. Since the overall outcomes of the infection on cellular metabolism may mask the actions of each viral protein individually, it is crucial to discriminate the effects of each protein separately to uncover new functions and potential targets for therapeutic interventions.

### Experimental procedures

#### Cell culture and infection

Huh7 cells, a human hepatocarcinoma cell line, were cultured in DMEM with 5 mM glucose (Gibco, USA), supplemented with 10% fetal bovine serum (FBS) (Invitrogen Corporation, USA), 100 U/mL penicillin, 100 g/mL streptomycin, 0.22% sodium bicarbonate, and 0.2% HEPES, pH 7.4, in a CO_2_ humid incubation chamber, at 37°C. When the cultures reached 70% confluence, the cells were infected with DENV serotype 2 (strain 16681), using an MOI of 1, or subjected to simulated infection (mock). After 24 h of infection, the cells were collected for mitochondria isolation or determination of NADH:ubiquinone oxidoreductase activity.

#### Isolation of mitochondria from DENV-infected HuH7 cells

Huh7 cells, seeded in 150 cm^2^ flasks (2 × 10^7^ cells), were infected with DENV (MOI = 1). After 24 h, the cells were collected and lysed with a Potter-Elvehjem homogenizer (Sigma-Aldrich, MO, USA). The cell lysate was centrifuged at 700 × *g*, for 10 min, at 4°C, to remove cell debris. The supernatant was collected and subsequently centrifuged at 15,000 × *g*, for 10 min, at 4°C, to obtain the enriched mitochondria preparation. To isolate mitochondria, we modified a protocol that separates mitochondria from other cellular compartments using a discontinuous sucrose gradient (35). Briefly, we reduced the rotation speed and compensated for this change with an increase in the centrifugation time. The enriched mitochondria preparation was deposited on a discontinuous sucrose gradient of 1.7 M under 1.0 M and centrifuged at 17,000 × *g*, for 80 min, at 4°C. The purity of the isolated mitochondria preparation was confirmed by western blotting (Fig. S4), using anti-mitofusin-1 (a mitochondrial protein) antibody (Abcam, Cambridge, UK) incubated overnight at 1:1,000 dilution, followed by anti-rabbit antibody IRDye800CW (LI-COR Biosciences, NE, USA) at 1:25,000 dilution for 1 h; or with anti-α-tubulin (a cytoplasmic protein) antibody (Merck Millipore, MA, USA) incubated at 1:10,000 dilution for 1 h, followed by anti-mouse antibody IRDye680RD (LI-COR Biosciences, NE, USA) incubated at 1:15,000 dilution for 1 h. The membrane was washed after the incubations and developed on Licor Odyssey Scanner (LI-COR Biosciences, NE, USA).

#### Proteomics screening

Mitochondria samples from infected Huh7 cells were incubated in a solution containing 0.2% of RapiGest (Waters, MA, USA) for 12 h, at 37°C under agitation (200 rpm) and submitted to sonication in the bath for 30 min, followed by centrifugation (12,000 rpm, for 1 min, at room temperature). Each sample was filtered and desalted using Centriprep of 3 kDa (Merck Millipore, MA, USA) and washed (three times) with 500 µL of NH_4_HCO_3_. The retained volume was dried using a Speed-Vac system, and the dried samples were resuspended in a solution containing 0.2% of RapiGest, followed by digestion steps according to the manufacturer’s instructions. The tryptic peptides, in triplicate, were loaded on a Waters nanoAcquity system (Waters, MA, USA). In total, 9 µL of the samples was injected (3 µL each time) and desalted online, using a Waters Symmetry C18 (180 µm × 20 mm, 5 µm trap column). By using a Waters ACQUITY UPLC Peptide HSS T3 C18 column (150 µm × 75 mm, 1.7 µm), a liquid chromatography step was performed using a 0.5 µL/min mobile phase flow with a linear gradient from 3% to 40% of acetonitrile containing 0.1% formic acid across a 210 min running time. Electrospray tandem mass spectra were recorded using a Waters Synapt G1 HD/MS High-Definition Mass Spectrometer (Waters, Manchester, UK) interfaced with the nanoAcquity system capillary chromatography. The parameters used during the experiments were: 3,000 V for ESI voltage, source temperature of 80°C, and cone voltage of 35 V. MassLynx data system (Version 4.1, Waters, MA, USA) was used for data acquisition and instrument control and performed by scanning from a mass-to-charge ratio (*m/z*) of 50–2,000, using a scan time of 1.0 s. MSE acquisition (Waters, Milford, MA, USA) was performed with collision energy alternating between 6 V in low energy and ramped from 12 to 40 V in high energy, using argon as the collision gas at a pressure of 1 bar. All data were processed using the Progenesis QI for proteomics version 2.0 software platform (Nonlinear Dynamics, Waters, Manchester, UK). The exact masses were determined using the Q-Tof’s LockSpray of GFP reference ion 785.8426 *m/z*. Raw data were searched in Progenesis against Databank_Human_DENV-strain_fasta (strain 16681) Uniprot non-reviewed protein database (downloaded in 2019, 40,734 sequences), including reversed sequences, human keratin proteins and *Sus scrofa* trypsin as possible contaminants. Cysteine carbamidomethylation was set as fixed modification, oxidation of methionine was set as variable modification, and peptide and fragment tolerance were set as 10 and 20 ppm, respectively, with the following ions requirements: two fragments peptide, five fragments protein, and one peptide. The false discovery rate was set to less than 1%, and the quantified proteins, with a *P* value lower than 0.05 for ANOVA, were considered a reliable identification for relative normalized abundance analysis.

#### NS3 expression and purification

*Escherichia coli* BL21 (DE3) was transformed with pET32a(+)_NS3pro (Biomatik, ON, Canada) containing residues 1–180 corresponding to the NS3 protease domain; or pET32a(+)_NS3proS135A, with the serine 135 of the catalytic triad replaced by alanine, which makes the protease inactive; or pET32a(+)_NS3prohel containing residues 1–618 corresponding to full-length NS3. Bacteria were grown in M9 minimal medium containing 100 µg/mL of ampicillin. Protein expression was induced with 0.6 mM IPTG when the optical density reached 0.6–0.7 at 600 nm. After 18 h of induction at 18°C, the cells were pelleted and resuspended in buffer A [50 mM Tris-HCl (pH 8), 500 mM NaCl, 20 mM imidazole, and 10% glycerol] and lysed by sonication. The lysate was centrifuged at 12,000 × *g*, for 60 min, at 4°C. Although part of the recombinant proteins was retained in inclusion bodies, the soluble fraction of the proteins was collected and purified from the supernatant. For this, the supernatant was filtered using a 0.22 µm filter, applied to HisTrap FF column (GE Healthcare Life Sciences, NJ, USA) and purified by immobilized metal affinity chromatography (IMAC) using the buffer A for equilibration and buffer B [50 mM Tris-HCl (pH 8), 500 mM NaCl, 500 mM imidazole, and 10% glycerol] for elution. Proteins were eluted with a 20–500 mM linear imidazole gradient. For NS3prohel purification, an additional gel filtration step was performed using a Sephacryl S-100 column (Cytiva, MA, USA). The eluates containing the recombinants proteins were placed in Amicon Ultra-4 3K centrifugal filter device (Merck Millipore, MA, USA), dialyzed with buffer C [50 mM Tris (pH 8), 150 mM NaCl, and 5% glycerol], and later with buffer D [50 mM Tris (pH 8), 5% glycerol, and 5% ethylene glycol]. The samples were then stored at −80°C and their purity confirmed by 15% SDS-PAGE (Fig. S1B and C).

#### NS3 activity

The activity of NS3pro or NS3S135A was performed using the fluorogenic substrate Bz-Arg-AMC·HCl (Santa Cruz Biotechnology, Dallas, USA), following an adapted protocol previously described (28). Briefly, the reaction was initiated by adding 1.8 µM NS3pro or NS3proS135A to the reaction mix [250 µM Bz-Arg-AMC·HCl, 200 mM Tris (pH 8.5)]. Blank samples were prepared by replacing the proteins’ preparations with the equivalent volume of H_2_O. The proteolytic activity was monitored for 45 min at 37°C with an excitation wavelength of 385 nm and an emission wavelength of 465 nm using a SpectraMax M5 (Molecular Devices, CA, USA). Relative fluorescence units (RFUs) were normalized by subtracting the blank values and subsequently by the protein content.

#### Isolation of mitochondria from mouse liver

Male C57BL/6 mice aged 12–20 weeks were euthanized by cervical dislocation, and the liver was quickly collected to initiate the mitochondria isolation. The experimental approaches were approved by the Committee on Ethics in Animal Use (CEUA) in Scientific Experimentation of the Health Sciences Center of the Federal University of Rio de Janeiro (protocol no. A39/23-046/22). Mitochondria isolation was performed using a method previously described (36), with some adaptations. Briefly, the tissue was lysed in isolation buffer [0.1 M Tris/MOPs (pH 7.4), 0.1 M EGTA/Tris (pH 7.4), 1 M sucrose] using Potter-Elvehjem homogenizer (Sigma-Aldrich, MO, USA) on ice. The homogenate was centrifuged at 700 × *g*, 4°C, 10 min, the supernatant was collected and centrifuged at 7,000 × *g*, at 4°C, for 10 min. The pellet containing mitochondria was washed in ice-cold isolation buffer and centrifuged at 8,500 × *g*, at 4°C, for 10 min. The protein concentration of the samples was evaluated by Bradford method. For all the experiments, the isolated mitochondria (300 µg protein) were previously incubated for 1 h with NS3pro, NS3proS135A, NS3prohel or, as a control, with the equivalent volume of buffer in which the recombinant proteins were diluted [50 mM Tris (pH 8), 5% glycerol, and 5% ethylene glycol] in MIR05 (22) supplemented with 2 mM ATP and 10 mM succinate, on ice.

#### NADH:ubiquinone oxidoreductase activity

NADH:ubiquinone oxidoreductase (CI) activity was performed using mitochondria isolated from mouse liver, incubated or not with NS3pro or NS3proS135A at different concentrations for 1 h, and subjected to freeze-thawing cycles to produce extracts following an adapted protocol previously described (37). The activity was evaluated in reaction buffer [50 mM potassium phosphate buffer (pH 7.5), 3 mg/mL fatty acid-free BSA, 300 µM KCN, and 100 µM NADH]. The reaction was initiated with 60 µM ubiquinone, and the absorbance of the samples was monitored for 2 min at 340 nm using a Shimadzu UV-1800 spectrophotometer (Shimadzu, Kyoto, Japan), followed by the addition of 10 µM rotenone (used to discount CI-independent NADH oxidoreductase activity). The calculation was carried out using the NADH extinction coefficient (6.2 mM⁻¹ cm⁻¹). We adapted the same protocol for DENV-infected Huh7 cells, using 80 µg of protein of cell extracts. Huh7 cells were collected 24 hpi, and the pellets were resuspended in 20 mM potassium phosphate buffer (pH 7.5). To lyse the cells, the suspension was taken up and expelled using a Hamilton syringe (Hamilton Company, NV, USA) until it became a homogeneous solution and subjected to freeze-thawing cycles.

#### Succinate dehydrogenase activity

The succinate dehydrogenase (CII) activity was performed with mouse liver mitochondria extracts incubated or not with NS3pro for 1 h, using an adapted protocol previously described (38). Briefly, the activity was determined using 2,6-dichlorophenolindophenol, DCPIP (Sigma-Aldrich, MO, USA), an artificial electron acceptor. The assay was conducted in 96-well plates using a SpectraMax M5 (Molecular Devices, CA, USA). Approximately 20 µg of isolated mitochondria were added to the wells and the reaction was started by adding 200 µL of reaction buffer [20 mM phosphate buffer (pH 7), 0.1% Triton X-100, 4 mM sodium azide, 5 mM succinate, and 300 mM DCPIP]. The plate was shaken for 10 s, and the DCPIP reduction was monitored for 3 min at 600 nm, 35°C. The activity calculation was performed using the reduced DCPIP absorption coefficient (21.0 mM^−1^ cm^−1^) and data represented normalized by the protein concentration of each sample.

#### Succinate:cytochrome *c* reductase activity (CII + CII)

CII + CIII activity was performed using mitochondria isolated from mouse liver, incubated or not with NS3pro for 1 h, following an adapted protocol previously described (37). The samples were incubated for 10 min in reaction buffer [50 mM potassium phosphate buffer (pH 7.5), 300 µM KCN, and 10 mM succinate], at 37°C. The reaction was started with 50 µM oxidized cytochrome *c*, and the cytochrome *c* reduction was monitored for 2 min at 550 nm using a Shimadzu UV-1800 spectrophotometer (Shimadzu, Kyoto, Japan), followed by the addition of 10 mM malonate (used to inhibit CII activity and discount background signal). The calculation was performed using the cytochrome *c* extinction coefficient (18.5 mM⁻¹ cm⁻¹) and normalized by the protein concentration of each sample.

#### Cytochrome c oxidase activity

Cytochrome *c* oxidase (CIV) activity was performed by high-resolution respirometry using the Oxygraph-2k system (Oroboros Instruments, Innsbruck, Austria) and DatLab 7.4.0.4 software (Oroboros Instruments, Innsbruck, Austria). The respirometry experiments were performed with mitochondria isolated from mouse liver (50 µg protein), incubated or not with NS3pro for 1 h, in a final volume of 2 mL of MIR05, at 37°C, stirring at 750 rpm. The oxygen concentration was monitored in the presence of 1 mM ascorbate and 1 mM TMPD, a CIV-specific electron donor, followed by the addition of 1.5 mM KCN (used to discount CIV-independent activity).

#### ATP synthase reverse activity

The ATP synthase (CV) reverse activity was based on ATP hydrolysis and subsequent determination of inorganic phosphate content. The activity was performed using mouse liver mitochondria extracts at 150 µg/mL protein concentration, incubated or not with NS3pro for 1 h, at 37°C. The reaction was started by adding 1 mL of reaction buffer (20 mM HEPES, 5 mM MgCl_2_, and 1 mM ATP) to the samples. For 30 min, aliquots were collected every 5 min, and the reaction was stopped with the addition of 20% TCA. Inorganic phosphate content was measured by the Fiske-Subbarow method (39) using a SpectraMax M5 (Molecular Devices, CA, USA). The same assay was conducted in the presence of 5 mM azide to inhibit ATP synthase and discount background signal.

#### High-resolution respirometry

Mitochondrial OCRs were assessed by high-resolution respirometry using the Oxygraph-2k system (Oroboros Instruments, Innsbruck, Austria) and DatLab 7.4.0.4 software (Oroboros Instruments, Innsbruck, Austria). The experiments were performed with isolated mitochondria from mouse liver (50 µg protein), incubated or not with NS3pro, NS3proS135A, or NS3prohel for 1 h, in a final volume of 2 mL of MIR05, at 37°C, stirring at 750 rpm. Different substrates and inhibitors were added in the following sequence and concentrations. For complexes I + III + IV-dependent respiration, 5 mM malate and 10 mM pyruvate were added, followed by 2.5 mM ADP, and then 0.25 µM rotenone. Respiratory state 2 represents the OCR after the addition of substrates (in this case malate and pyruvate) but in the absence of ADP. Respiratory state 3 represents the OCR value reached after ADP addition. Rotenone was added to determine the residual rate of non-mitochondrial oxygen consumption, which is subtracted from OCR values for the analyses. For the multisubstrate protocol, 5 mM malate, 10 mM pyruvate, and 10 mM succinate were added, followed by 2.5 mM ADP, and then 0.25 µM rotenone and 0.5 mM antimycin. Leak respiration, a non-phosphorylating resting state when substrates are oxidized but ADP is not supplied, was calculated by subtracting residual oxygen consumption (in the presence of rotenone and antimycin) from the respiratory state 2.

#### Mitochondrial membrane potential

The mitochondrial membrane potential (Δψm) was analyzed using safranin O (Sigma-Aldrich, MO, USA). This lipophilic cationic dye self-quenches its fluorescence through its potential-dependent distribution between the external environment and the intramitochondrial compartment. The assay was carried out in a Varian Cary Eclipse fluorometer (Agilent, CA, USA), with an excitation wavelength of 495 nm, an emission wavelength of 587 nm and high-speed stirring at 37°C. The cuvettes were prepared with 1 mL of MIR05, isolated mitochondria (50 µg protein) incubated or not with NS3pro for 1 h, and 0.5 µM safranin O. The assay started with the addition of 5 mM malate, 10 mM pyruvate, and 10 mM succinate, followed by 200 µM ADP, 0.1 µg/mL oligomycin and 0.05 µM FCCP. The maximum membrane potential was calculated by subtracting the value of safranin O fluorescence intensity obtained after oligomycin addition (hyperpolarization) from the minimum fluorescence intensity obtained after the depolarization with FCCP. The percentage of the maximum mitochondrial membrane potential maintained in the phosphorylating state was obtained by calculating the difference between the fluorescence value obtained after ADP addition and after FCCP addition, divided by the maximum membrane potential, and then multiplied by 100.

#### Prediction of viral protease cleavage sites in CI subunits

The presence of viral protease cleavage sites in the mitochondrial CI subunits was evaluated using the SitePrediction website (https://www.dmbr.ugent.be/prx/bioit2-public/SitePrediction/) (24). The searches were performed by entering the 12 combinations of amino acids that create potential cleavage sites at positions P2-P1xP1′ (KRA, KRG, KRS, RRA, RRG, RRS, RKA, RKG, RKS, QRA, QRG, and QRS), considering that the NS2B-NS3 cleavage motifs present in flavivirus polyproteins consist of a pair of basic amino acid residues (KR, RR, RK, or occasionally QR) at the canonical positions P2 and P1 followed by a small amino acid (G, S, or A) in position P1′ (20, 25). We entered the protein sequences of the 44 subunits from mouse or human CI as substrate inputs. Only sites with 100% similarity to the researched sites were considered for each substrate sequence. The potential cleavage sites in CI were represented in the complex structure (PDB: 5XTD) using PyMOL.

#### Statistical analysis

Statistical analyses were performed using GraphPad Prism 8.0.1 (GraphPad Software, CA, USA) and data were expressed as mean ± standard deviation. Differences between means were analyzed using one-way ANOVA with Sidak’s post hoc test or Student’s *t* test. Differences with *P* < 0.05 were considered statistically significant.

## Data Availability

The mass spectrometry proteomics data contained in this paper are publicly available through MassIVE repository (doi:10.25345/C5ZS2KQ29). All other original data reported in this paper will be shared by the lead contact upon request.

## SUPPLEMENTAL MATERIAL

Supplemental figures and tables msphere.00406-24-s0001.pdf Table S1 and Figures S1 to S4.

ASM does not own the copyrights to Supplemental Material that may be linked to, or accessed through, an article. The authors have granted ASM a non-exclusive, world-wide license to publish the Supplemental Material files. Please contact the corresponding author directly for reuse.
