# Supplementary material for: Dengue virus non-structural protein 3 inhibits mitochondrial respiration by impairing complex I function
Source: mSphere. 2024 Jul 9;9(7):e00406-24. doi: 10.1128/msphere.00406-24 (PMC11288018; doi:10.1128/msphere.00406-24)
Supplement: Supplemental figures and tables — Table S1 and Figures S1 to S4. [file msphere.00406-24-s0001.pdf]

Dengue virus non-structural protein 3 inhibits mitochondrial respiration by impairing complex I function

Supplementary information

Table S1. Differentially expressed proteins in isolated mitochondria from DENV-infected Huh7 cells, ordered by fold change.

Protein name	Accession	Peptide count	Unique peptides	Fold change decrease relative to mock
Mitochondrial pyruvate carrier 1	Q9Y5U8	1	1	Infinity
Erlin-1	O75477	3	1	9,419946815
Beta-actin-like protein 2	Q562R1	5	1	5,331133383
Putative heat shock protein HSP 90-alpha A5	Q58FG0	8	1	3,981651002
Oligosaccharyltransferase complex subunit OSTC	Q9NRP0	1	1	3,65329968
NADH dehydrogenase (ubiquinone) 1 alpha subcomplex subunit 9, mitochondrial *	Q16795	2	2	3,609761243
Ras-related protein Rab-10	P61026	6	1	3,474165322
Surfeit locus protein 4	O15260	4	2	3,243158327
Amine oxidase [flavin-containing] B	P27338	2	2	3,043336011
RNA-binding protein Musashi homolog 2	Q96DH6	1	1	2,834187272
Mitochondrial carrier homolog 2	Q9Y6C9	5	5	2,747435429
Tubulin alpha-1C chain	Q9BQE3	13	1	2,617916804
Erlin-2	O94905	6	3	2,583379753
Mitochondrial import receptor subunit TOM22 homolog	Q9NS69	2	2	2,566999952
Very-long-chain (3R)-3-hydroxyacyl-CoA dehydratase 3	Q9P035	6	4	2,472867908
Calcium load-activated calcium channel	Q9UM00	3	2	2,472185745
Uridine diphosphate glucose pyrophosphatase NUDT14	O95848	1	1	2,400975718
ATP synthase subunit f, mitochondrial *	P56134	4	3	2,246875441
Receptor expression-enhancing protein 6	Q96HR9	1	1	2,241302476
Dolichyl-diphosphooligosaccharide--protein glycosyltransferase subunit 2	P04844	19	17	2,224549122
Leucine-rich repeat-containing protein 59	Q96AG4	14	12	2,121383016
Cytochrome b-c1 complex subunit 9 *	Q9UDW1	1	1	2,081254815
ATP synthase membrane subunit K, mitochondrial *	Q96IX5	2	1	2,065188135
MICOS complex subunit MIC60	Q16891	18	14	2,053471114
Dolichyl-diphosphooligosaccharide--protein glycosyltransferase 48 kDa subunit	P39656	17	15	2,004083047

* Belongs to the mitochondrial electron transport system (ETS).

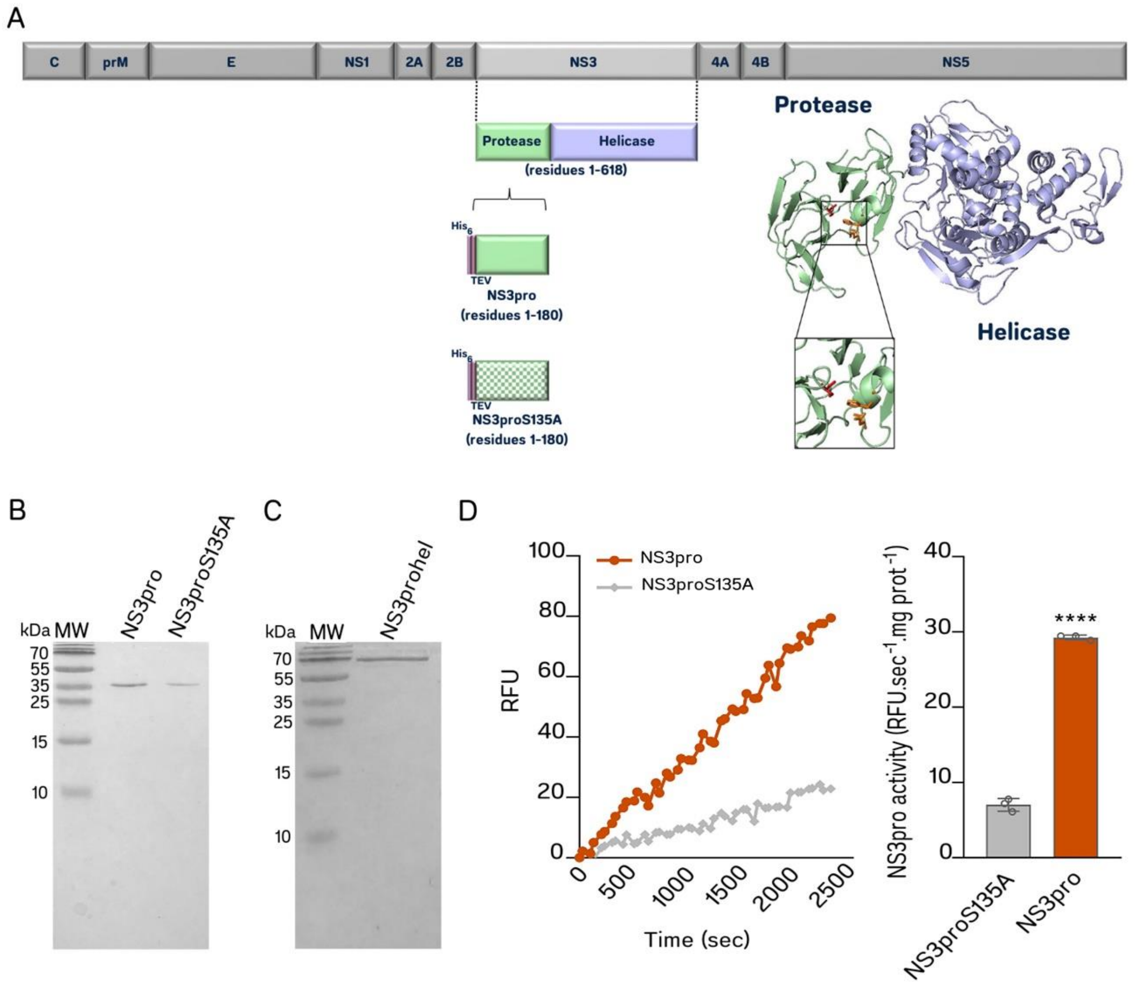

Figure S1: Recombinant proteins produced for the assays. (A) Scheme of the DENV polyprotein highlighting the NS3 peptide and the constructs used in this study. The recombinant NS3pro construct comprises residues 1-180 (green) of DENV NS3 protein and has a hexa-histidine tag (His-tag) (lilac) and a TEV cleavage sequence (purple). The ribbon representation of NS3 protein with the helicase (blue) and protease (green) domains, highlighting the catalytic triad composed of histidine at position 51 (orange), aspartate at position 75 (orange), and serine at position 135 (red), was generated with PyMOL using PDB ID: 2VBC. NS3proS135A (green checkered) is catalytically inactive due to a substitution of serine 135 for an alanine. (B) SDS-PAGE of recombinant NS3pro and NS3proS135A proteins. (C) SDS-PAGE of recombinant NS3prohel protein. MW – molecular weight standard. (D) Enzymatic activity of recombinant NS3pro and NS3proS135A using a fluorogenic peptide as substrate. On the left are representative kinetics in the different conditions. On the right is a plot with the reaction rates obtained in independent experiments (n=3). Data expressed as mean ± standard deviation. Significant differences between means were analyzed using Student's t-test. ****, p<0,0001.

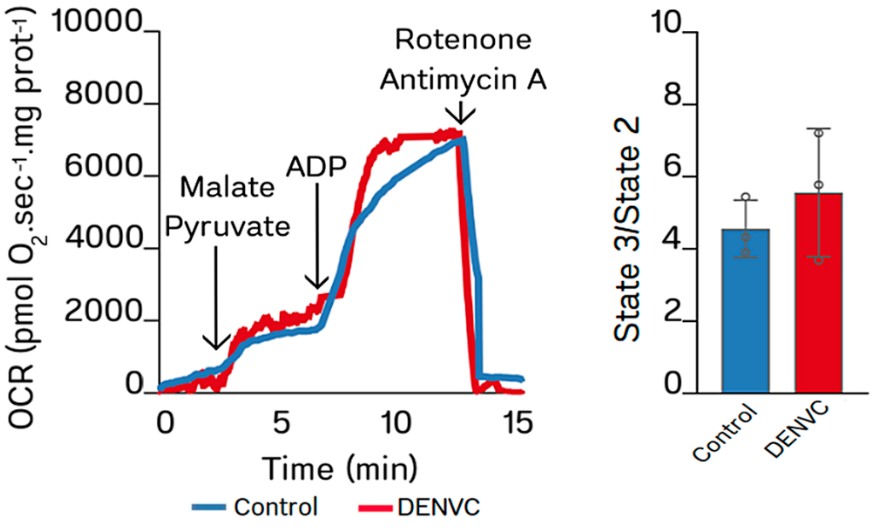

Figure S2: DENV capsid protein does not affect malate/pyruvate oxidation. High-resolution respirometry experiments with isolated mitochondria incubated with DENV capsid protein (DENVC) using pyruvate and malate as substrates, followed by the addition of ADP and inhibitors, as indicated. The graph on the left shows a representative high-resolution respirometry experiment, and the graph on the right shows a fold change of respiratory state 3 relative to respiratory state 2 obtained in independent experiments (n=3). The residual non-mitochondrial oxygen consumption rate was discounted from the analysis. The control condition is represented in blue, and mitochondria samples incubated with DENVC are represented in dark red. Data expressed as mean ± standard deviation. No significant differences were observed when comparing the means using Student's t-test.

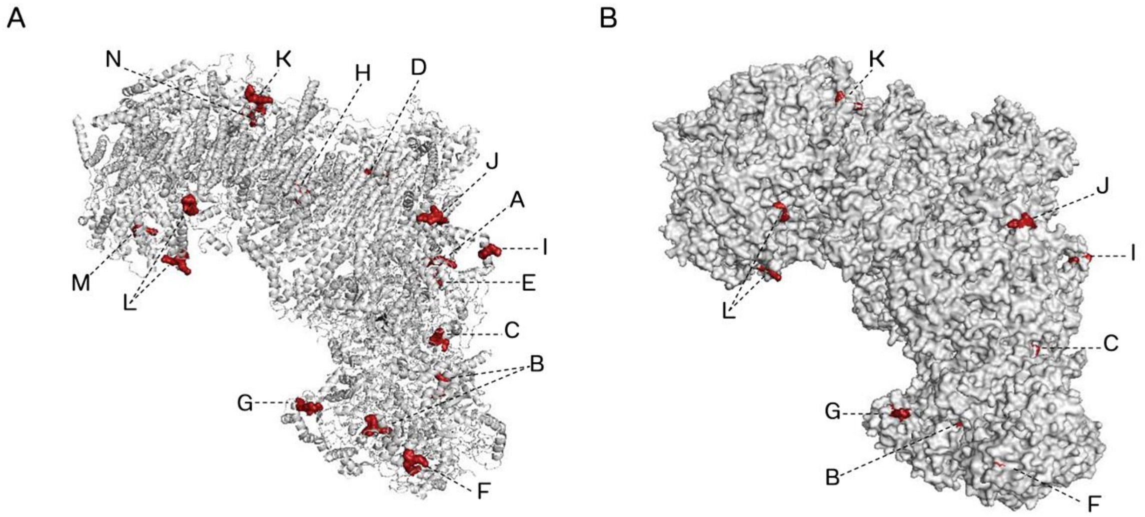

Figure S3: Predicted NS3 protease cleavage sites in mitochondrial CI subunits. (A) Ribbon diagram of human CI structure (PDB: 5XTD, in gray) with the predicted NS3 cleavage sites highlighted in red. (B) Surface representation of the CI structure highlighting the potential NS3 cleavage sites that are more exposed to the surface. To facilitate correspondence, here the potential NS3 cleavage sites were identified with the same letters as in Table 2 in the main text (A-N).

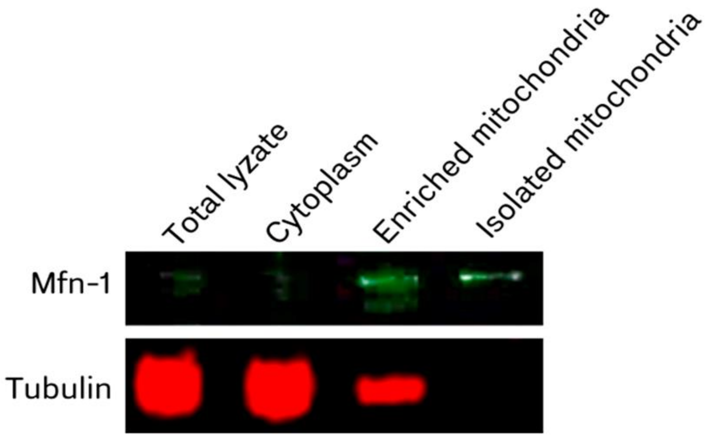

Figure S4: Testing the purity of isolated mitochondria samples (prepared by saccharose gradient) from Huh7 cells used in the proteomics screening. Western blotting of fractions collected during the sample processing for mitochondrial isolation from Huh7 cells. The total lysate (first lane) corresponds to the first step of the processing after cells were lysed mechanically. The cytoplasm fraction (second lane) corresponds to the supernatant after the samples were centrifuged to form a pellet enriched in mitochondria (third lane). The isolated mitochondria fraction was obtained after the enriched mitochondria preparation was further purified in a saccharose gradient. In green, mitofusin-1 (Mfn-1) was used as a mitochondrial marker; in red, tubulin was used as a cytoplasmic marker.
